# Development of early prediction model of in-hospital cardiac arrest based on laboratory parameters

**DOI:** 10.1186/s12938-023-01178-9

**Published:** 2023-12-06

**Authors:** Xinhuan Ding, Yingchan Wang, Weiyi Ma, Yaojun Peng, Jingjing Huang, Meng Wang, Haiyan Zhu

**Affiliations:** 1 Medical School of Chinese PLA, Beijing, 100853 China; 2 Department of Emergency, The First Medical Center, Chinese PLA General Hospital, Fuxing Road 28, Beijing, 100853 People’s Republic of China; 3 The Second School of Clinical Medicine, Southern Medical University, Guangzhou, 510000 Guangdong China; 4 Department of Emergency, Hainan Hospital of PLA General Hospital, Sanya, 572013 Hainan China

**Keywords:** Algorithm, In-hospital cardiac arrest (IHCA), Prediction model, Laboratory parameters, Coagulation, Rescue treatment

## Abstract

**Background:**

In-hospital cardiac arrest (IHCA) is an acute disease with a high fatality rate that burdens individuals, society, and the economy. This study aimed to develop a machine learning (ML) model using routine laboratory parameters to predict the risk of IHCA in rescue-treated patients.

**Methods:**

This retrospective cohort study examined all rescue-treated patients hospitalized at the First Medical Center of the PLA General Hospital in Beijing, China, from January 2016 to December 2020. Five machine learning algorithms, including support vector machine, random forest, extra trees classifier (ETC), decision tree, and logistic regression algorithms, were trained to develop models for predicting IHCA. We included blood counts, biochemical markers, and coagulation markers in the model development. We validated model performance using fivefold cross-validation and used the SHapley Additive exPlanation (SHAP) for model interpretation.

**Results:**

A total of 11,308 participants were included in the study, of which 7779 patients remained. Among these patients, 1796 (23.09%) cases of IHCA occurred. Among five machine learning models for predicting IHCA, the ETC algorithm exhibited better performance, with an AUC of 0.920, compared with the other four machine learning models in the fivefold cross-validation. The SHAP showed that the top ten factors accounting for cardiac arrest in rescue-treated patients are prothrombin activity, platelets, hemoglobin, N-terminal pro-brain natriuretic peptide, neutrophils, prothrombin time, serum albumin, sodium, activated partial thromboplastin time, and potassium.

**Conclusions:**

We developed a reliable machine learning-derived model that integrates readily available laboratory parameters to predict IHCA in patients treated with rescue therapy.

**Supplementary Information:**

The online version contains supplementary material available at 10.1186/s12938-023-01178-9.

## Background

In-hospital cardiac arrest (IHCA) is an acute disease with a high fatality rate that burdens individuals, society, and the economy [1, 2]. There are approximately 290,000 cases of IHCA in the United States annually, with only 25% of such cases surviving and being discharged from the hospital [3]. A study [4] of IHCA in China observed that the incidence of IHCA was 17.5 per 1000 admissions, and the rates of return of spontaneous circulation and survival to hospital discharge were 35.5% and 9.1%, respectively. Thus, the current situation of IHCA in China is still concerning. Although technologies, such as mild hypothermia and extracorporeal membrane oxygenation, are increasingly used in cardiac arrest therapy [5, 6], patient prognosis remains poor. Therefore, identifying patients with high risk of IHCA is crucial for early intervention.

Machine learning has been demonstrated as a powerful tool that could detect unnoticed data trends and patterns in the use of conventional statistical models [7]. Recently, machine learning methods are increasingly applied to predict IHCA in hospitalized patients, emergency department patients, and intensive care unit patients [8–11]. However, to the best of our knowledge, a few studies explored approaches for predicting IHCA in patients treated with rescue therapy. Compared with previous study patients, patients treated with rescue therapy are in worse condition and need more efficient risk assessment. Furthermore, laboratory parameter are subject to strict quality control and have been identified as independent risk factors of poor patient outcomes [12, 13]. However, previous studies mainly focused on the performance of the model, with limited emphasis on the predictive factors [14–16]. Moreover, most previous studies only included blood cell counts and biochemical markers but overlooked the predictive value of coagulation markers [9, 11, 17, 18]. A study conducted by Deng et al. reported that D-dimer was associated with immediate mortality in patients with IHCA, while other markers related to coagulation were not analyzed [19]. Meanwhile, a number of former studies included subjective and unstructured variables in prediction modeling, which need manual discrimination or data conversion that might not be applicable to a rescue setting [8, 11, 20–23]. Laboratory results are objective and readily available; however, no previous studies have used machine learning to predict cardiac arrest solely based on routine laboratory parameters.

Therefore, we aimed to develop an appreciable model solely using routine laboratory data obtained from hospital information system (including blood counts, biochemical markers, and coagulation markers) to predict incident IHCA in patients requiring rescue therapy.

## Results

### Population characteristics

**Fig. 1:**
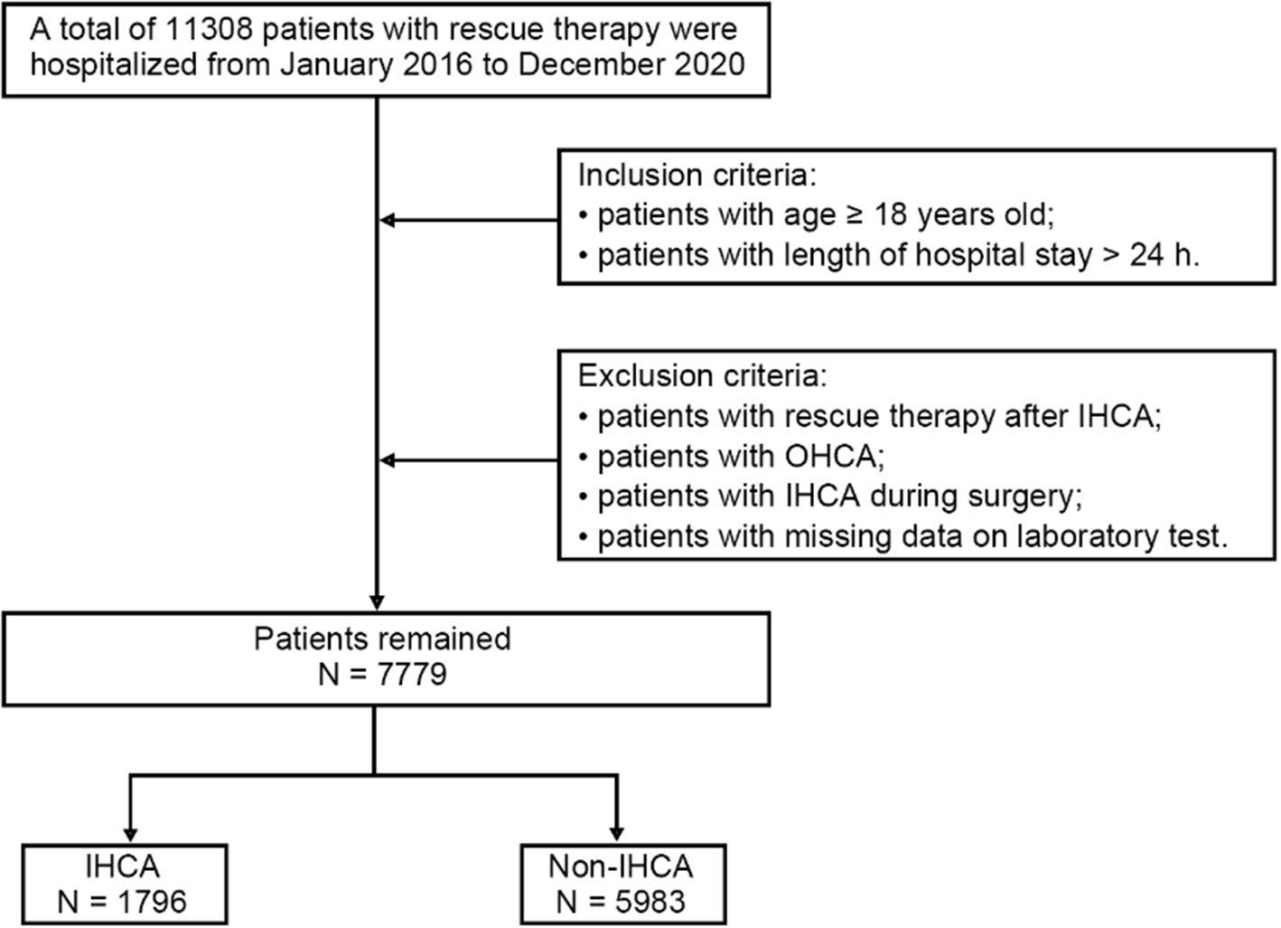
The screening phase flowchart. *OHCA* out-of-hospital cardiac arrest, *IHCA* in-hospital cardiac arrest

A total of 11,308 hospitalized patients receiving rescue therapy were included in this study, and 3529 patients were excluded according to the exclusion criteria. Among 7779 patients included in the present analysis, 1796 patients experienced IHCA (positive samples) and 5983 patients did not experience IHCA (negative samples) (Fig. 1). The incidence rate of IHCA did not differ by sex (63.73% vs 64.70%, *P* = 0.453). Patients with IHCA were older (80 years old vs 63 years old, *P* < 0.001) and had a higher proportion of comorbidities, such as hypertension (48.55% vs 38.84%, *P* < 0.001) and diabetes (25.84% vs 21.16%, *P* < 0.001), compared with those without IHCA (Table 1).

**Table 1:** Baseline characteristics of the non-IHCA cohort and the IHCA cohort

Characteristic mean (SD) or *N* (%)	Non-IHCA (*n* = 5983)	IHCA (*n* = 1796)	*P* value
Age, years	63 (51–73)	80 (65–87.25)	< 0.001
Gender = male	3813(63.73)	1162 (64.70)	0.453
BMI, kg/m^2^	24 (21–27)	22 (19–25)	< 0.001
Smoking	1691 (28.26)	416 (23.16)	< 0.001
Drinking	1610 (26.91)	342 (19.04)	< 0.001
Complications			
Hypertension	2324 (38.84)	872 (48.55)	< 0.001
Diabetes	1266 (21.16)	464 (25.84)	< 0.001
Laboratory results			
d-Dimer, ug/ml	1.18 (0.44–2.99)	3.51 (2.03–7.11)	< 0.001
Sodium, mmol/l	140.4 (137.6–142.6)	142.2 (136.8–148.8)	< 0.001
White blood cell count, 10^9^/l	7.23 (5.41–10.27)	11.9 (7.34–17.71)	< 0.001
Direct bilirubin, umol/l	3.9 (2.6–6.62)	10.8 (5–34.4)	< 0.001
PT, s	14 (13.2–15.3)	19.1 (16.1–24.4)	< 0.001
PTA, %	86 (73–97)	50 (35–67)	< 0.001
TT, s	15.9 (15–16.9)	17.1 (15.5–20.55)	< 0.001
Phosphorous, mmol/l	1.07 (0.89–1.25)	1.19 (0.83–1.72)	< 0.001
Creatinine, umol/l	75.9 (60.9–97.2)	132.2 (75.7–230.3)	< 0.001
Lumbar disc herniation, U/l	191.5 (152.78–294.3)	391.35 (250.88–741.48)	< 0.001
Alanine aminotransferase, U/l	17.7 (11.1–32.9)	20.65 (9.3–56.98)	< 0.001
Potassium, mmol/l	3.92 (3.64–4.23)	4.28 (3.81–4.85)	< 0.001
NT-proBNP, pg/ml	414.25 (114.2–1797.75)	5006 (1462.75–12,468.25)	< 0.001
Magnesium, mmol/l	0.86 (0.79–0.92)	0.88 (0.75–1)	< 0.001
Glucose, mmol/l	6.06 (4.94–8.15)	8.56 (6.47–12.07)	< 0.001
Platelets, 10^9^/l	198 (151–255)	92 (40–167)	< 0.001
Amylase, U/l	53.6 (37.7–79.18)	71.2 (38.98–136.5)	< 0.001
Hemoglobin, g/l	119 (98–136)	87 (73–104)	< 0.001
Calcium, mmol/l	2.17 (2.05–2.26)	2.05 (1.88–2.18)	< 0.001
APTT, s	37.9 (34.2–42.9)	49.4 (42–62.08)	< 0.001
Hematocrit, L/l	0.35 (0.29–0.4)	0.26 (0.22–0.32)	< 0.001
Fibrinogen, g/l	3.54 (2.84–4.6)	2.92 (1.9–4.18)	< 0.001
Neutrophils	0.72 (0.61–0.86)	0.88 (0.8–0.93)	< 0.001
Total protein, g/l	64.4 (58.3–69.5)	57.1 (51.1–63.3)	< 0.001
Troponin T, ng/ml)	0.02 (0.01–0.13)	0.11 (0.05–0.28)	0.009
Serum uric acid, umol/l	302.1 (230.6–388.48)	387.9 (249.55–562.35)	0.001
Chloride, mmol/l	103.1 (100.1–105.9)	102.4 (97–108.7)	0.001
Serum albumin, g/l	37 (32.2–40.6)	31 (27.4–34.7)	< 0.001
Aspartate aminotransferase, U/l	20.1 (14.4–39)	42.95 (21–125.45)	< 0.001
CRP, mg/dl	1.31 (0.21–5.7)	6.77 (3.03–12.13)	< 0.001

*BMI* Body Mass Index, *PT* prothrombin time, *PTA* prothrombin activity, *TT* thrombin time, *NT-proBNP* N-terminal pro-BNP, *APTT* activated partial thromboplastin time, *CRP* C-reactive protein

After undersampling, 1796 patients with IHCA and 1796 patients without IHCA were included in the model development. Basic characteristics are shown in Additional file 2: Table S1. After randomly grouping at a ratio of 8:2, the training set included 2873 patients and the testing set included 719 patients. The basic characteristics of the training set and testing set are listed in Table 2. There was no statistically significant difference between training set and testing set.

**Table 2:** Baseline characteristics of the undersampled training cohort and the testing cohort

Characteristic mean (SD) or *N* (%)	Training (*n* = 2873)	Testing (*n* = 719)	*P* value
Age, years	70 (57–83)	71 (57–83)	0.839
Male	1838 (63.97)	455 (63.28)	0.730
BMI, kg/m^2^	23 (20–26)	24 (21–26)	0.060
Smoking	759 (26.42)	184 (25.59)	0.652
Drinking	688 (23.95)	157 (21.84)	0.233
Complications			
Hypertension	1268 (44.14)	333 (46.31)	0.293
Diabetes	677 (23.56)	166 (23.09)	0.787
Laboratory results			
d-Dimer, ug/ml	2.22 (0.83–4.85)	2.3 (0.81–4.6)	0.635
Sodium, mmol/l	140.9 (137.1–144.6)	141.1 (137.8–145.1)	0.601
White blood cell count, 10^9^/l	8.91 (5.95–14.01)	8.81 (5.96–13.25)	0.286
Direct bilirubin, umol/l	5.8 (3.1–14.6)	5.9 (3.1–14.7)	0.011
PT, s	15.2 (13.7–18.7)	15.4 (13.8–19.5)	0.348
PTA, %	74 (52–90)	72 (49–90)	0.203
TT, s	16.2 (15.2–17.8)	16.2 (15–18.1)	0.441
Phosphorous, mmol/l	1.1 (0.86–1.38)	1.13 (0.88–1.47)	0.059
Creatinine, umol/l	88.2 (64.35–163.2)	86.4 (63. 4–155.6)	0.187
Lumbar disc herniation, U/l	259.5 (173.1–483.8)	281 (176.6–520.55)	0.552
Alanine aminotransferase, U/l	18.2 (10.6–40.9)	19.75 (10.38–40.65)	0.203
Potassium, mmol/l	4.04 (3.71–4.51)	4.04 (3.71–4.52)	0.886
NT-proBNP, pg/ml	1762 (379.7–6683.25)	1732 (284.82–7611.5)	0.637
Magnesium, mmol/l	0.86 (0.77–0.95)	0.87 (0.79–0.96)	0.109
Glucose, mmol/l	7.24 (5.36–10.09)	7.18 (5.35–10.41)	0.652
Platelets, 10^9^/l	160 (85–226)	160 (78–225)	0.371
Amylase, U/l	60.3 (38.4–106.4)	62.6 (38.28–110.5)	0.414
Hemoglobin, g/l	102 (82–126)	100 (80–125)	0.835
Calcium, mmol/l	2.11 (1.96–2.24)	2.12 (1.95–2.23)	0.496
APTT, s	40.8 (36.1–50.3)	41.1 (35.5–52.28)	0.326
Hematocrit, L/l	0.31 (0.25–0.37)	0.31 (0.24–0.37)	0.436
Fibrinogen, g/l	3.35 (2.47–4.46)	3.4 (2.51–4.42)	0.807
Neutrophils	0.83 (0.68–0.9)	0.83 (0.69–0.9)	0.640
Total protein, g/l	61 (53.9–67)	61.1 (53.6- 67.5)	0.820
Troponin T, ng/ml)	0.06 (0.02–0.23)	0.08 (0.02–0.23)	0.479
Serum uric acid, umol/l	327.7 (235.25–467.75)	331.7 (234.65–458.1)	0.911
Chloride, mmol/l	102.9 (98.8–106.8)	103.2 (99.1–107.1)	0.940
Serum albumin, g/l	33.7 (29.2–38.3)	34.2 (29.02–38.7)	0.633
Aspartate aminotransferase, U/l	27 (16–74.9)	28.1 (16.2–73.7)	0.264
CRP, mg/dl	3.94 (0.88–9.8)	4.75 (0.84–10.09)	0.277

*BMI* Body Mass Index, *PT* prothrombin time, *PTA* prothrombin activity, *TT* thrombin time, *NT-proBNP* N-terminal pro-BNP, *APTT* activated partial thromboplastin time, *CRP* C-reactive protein

### Model development and validation

**Fig. 2:**
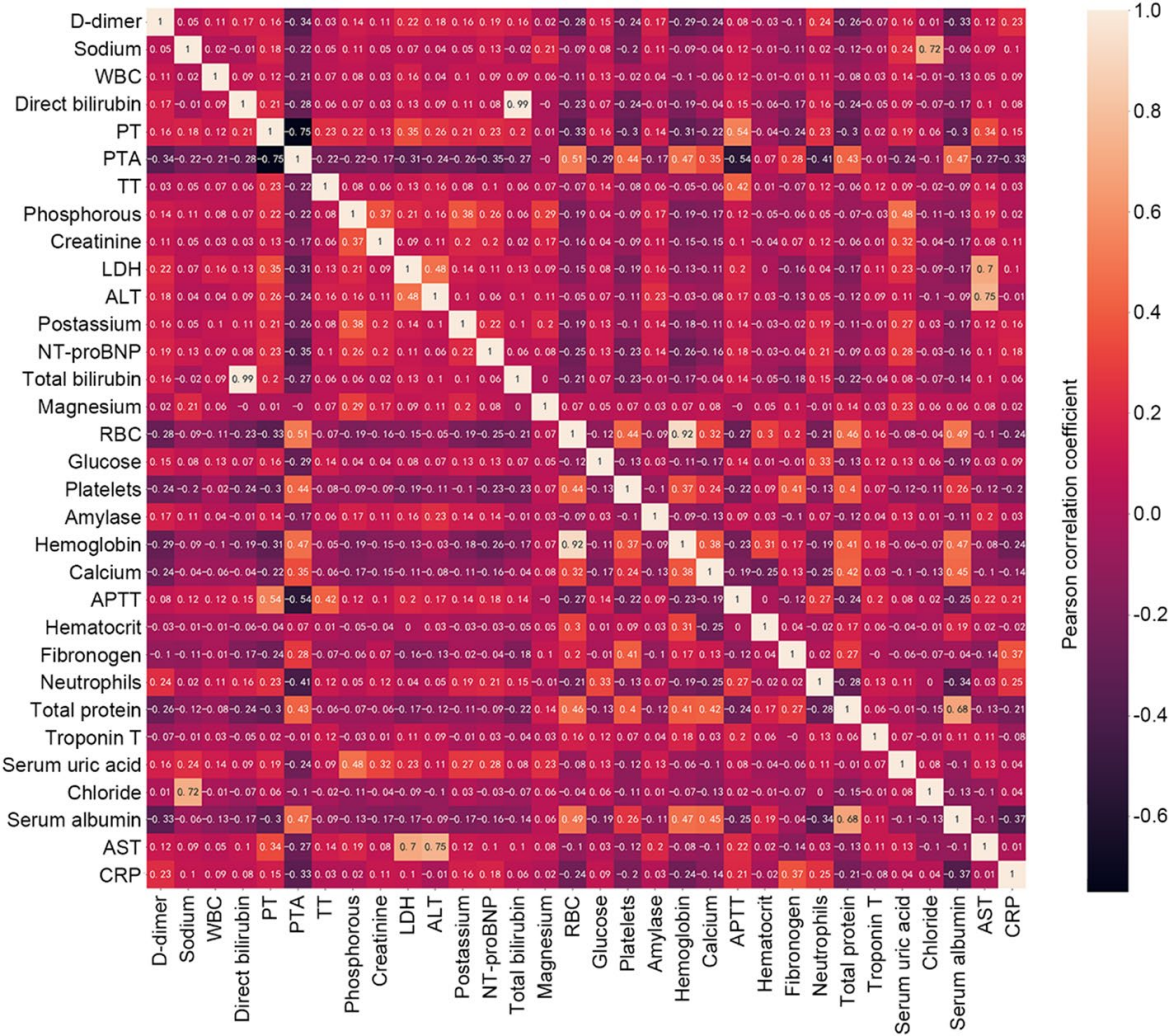
Correlation between variables. *WBC* white blood cell, *PT* prothrombin time, *PTA* prothrombin activity, *TT* thrombin time, *LDH* lumbar disc herniation, *ALT* alanine aminotransferase, *NT-proBNP* N-terminal pro-BNP, *RBC* red blood cells, *APTT* activated partial thromboplastin time, *TnT* troponin T, *AST* aspartate aminotransferase, *CRP* C-reactive protein

We used a heatmap to present the correlation coefficient between all variables (Fig. 2), and the results showed that hemoglobin and red blood cell, direct bilirubin, and total bilirubin had high correlation coefficient (> 0.8), respectively. The variable importance ranked by extra trees classifier (ETC) algorithm showed that red blood cell and total bilirubin had lower variable importance compared with their counterparts. Therefore, we discarded red blood cell and total bilirubin in the model development.

**Fig. 3:**
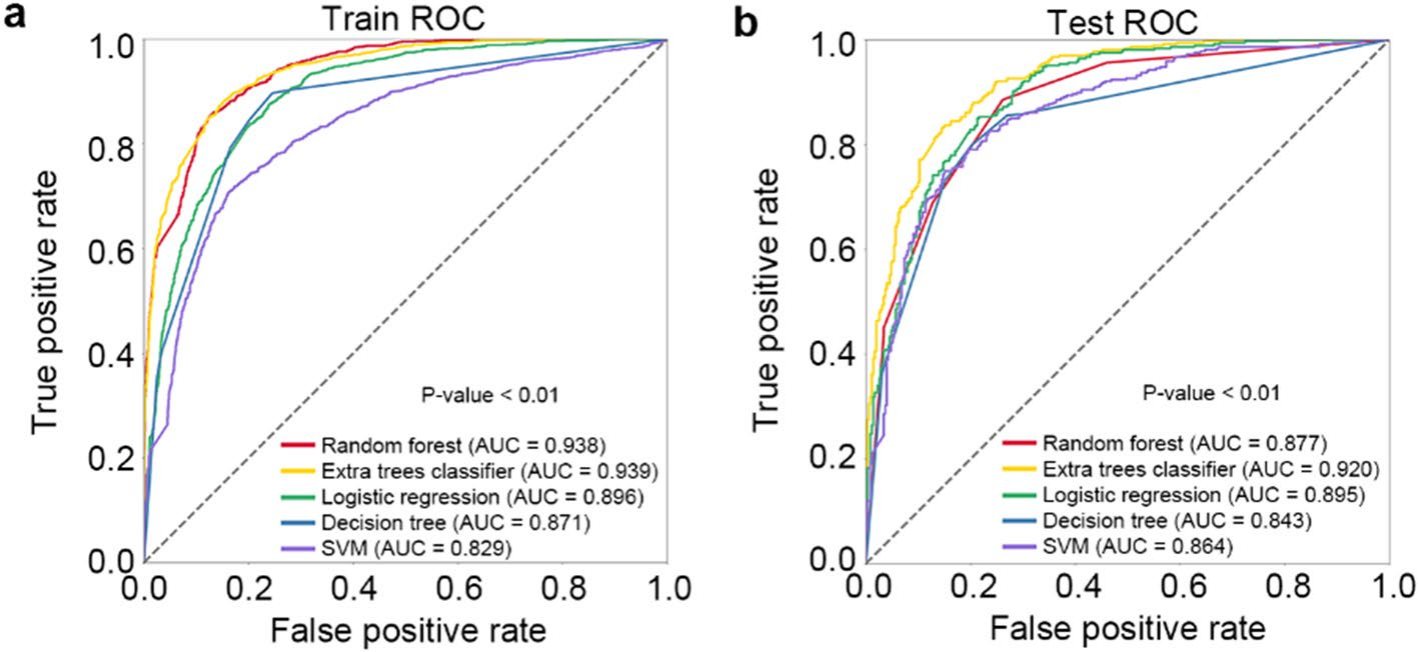
The ROC curve of different machine learning algorithms predicting IHCA in the training group and testing group. *ROC* receiver-operating characteristic, *SVM* support vector machine

**Fig. 4:**
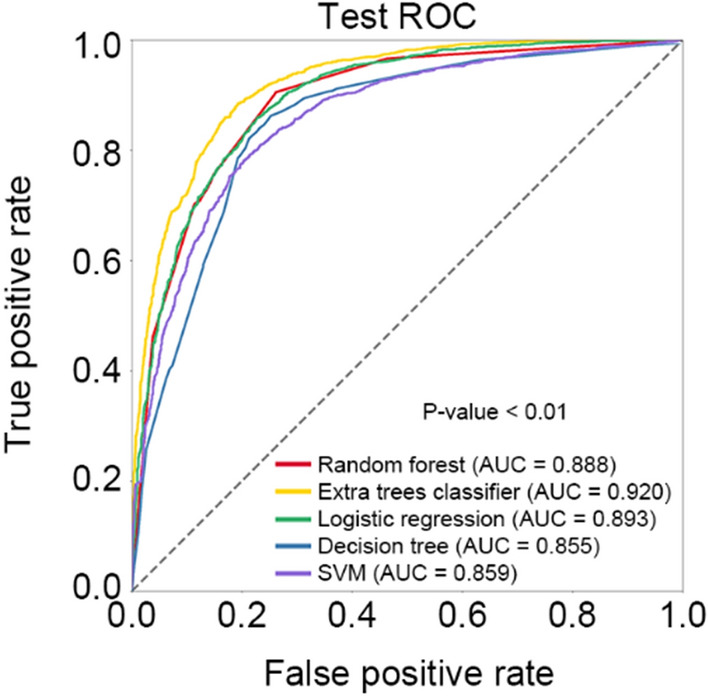
The ROC curve of different machine learning algorithms predicting the IHCA in the fivefold cross-validation. *ROC* receiver-operating characteristic, *SVM* support vector machine

ETC, logistic regression, random forest, support vector machine (SVM), and decision tree algorithms were used to develop the prediction models of IHCA in patients treated with rescue therapy. In the training group, we observed that ETC algorithm showed better performance compared with the other four algorithms (area under curve [AUC], 0.939 in ETC vs. 0.896 in logistic regression, 0.938 in random forest, 0.829 in SVM, and 0.871 in decision tree; *P* for comparison < 0.01) (Table 3 and Fig. 3). In the testing group, ETC algorithm also showed the best performance among five algorithms (AUC, 0.920 in ETC vs. 0.895 in logistic regression, 0.877 in random forest, 0.864 in SVM, and 0.843 in decision tree; *P* for comparison < 0.01) (Table 3 and Fig. 3). After fivefold cross-validation, the ETC algorithm generally provided the best overall performance regarding the AUC, accuracy, specificity, sensitivity, and F1-score compared with the other four algorithms (Table 3 and Fig. 4), indicating the consistency and robustness of our model. After considering these scores, especially the AUCs, we chose ETC model as the final prediction model. The hyperparameters for the ETC model as selected are summarized as follows: number of trees (n_estimators) = 100, supported criteria (criterion) = gini, maximum tree depth (max_depth) = 12, minimum number of samples leaf (min_samples_leaf) = 1, and minimum number of samples split (min_samples_split) = 2.

**Table 3:** Summary of model performance of five machine learning algorithms

Model	AUC	Accuracy	Specificity	Sensitivity	F1-score
Training set					
Extra trees classifier	0.939	0.858	0.829	0.901	0.863
Logistic regression	0.896	0.806	0.840	0.753	0.794
Random forest	0.938	0.853	0.825	0.894	0.858
Support vector machine	0.829	0.511	0.504	0.987	0.667
Decision tree	0.871	0.825	0.783	0.896	0.836
Testing set					
Extra trees classifier	0.920	0.834	0.818	0.869	0.843
Logistic regression	0.895	0.807	0.841	0.766	0.802
Random forest	0.877	0.784	0.831	0.725	0.774
Support vector machine	0.864	0.542	0.527	0.995	0.689
Decision tree	0.843	0.794	0.768	0.856	0.809
Fivefold cross-validation					
Extra trees classifier	0.920	0.841	0.787	0.895	0.849
Logistic regression	0.894	0.808	0.816	0.798	0.805
Random forest	0.889	0.803	0.853	0.754	0.792
Support vector machine	0.867	0.570	0.156	0.985	0.697
Decision tree	0.848	0.806	0.753	0.858	0.816

*AUC*, area under the curve

### Model interpretation

**Fig. 5:**
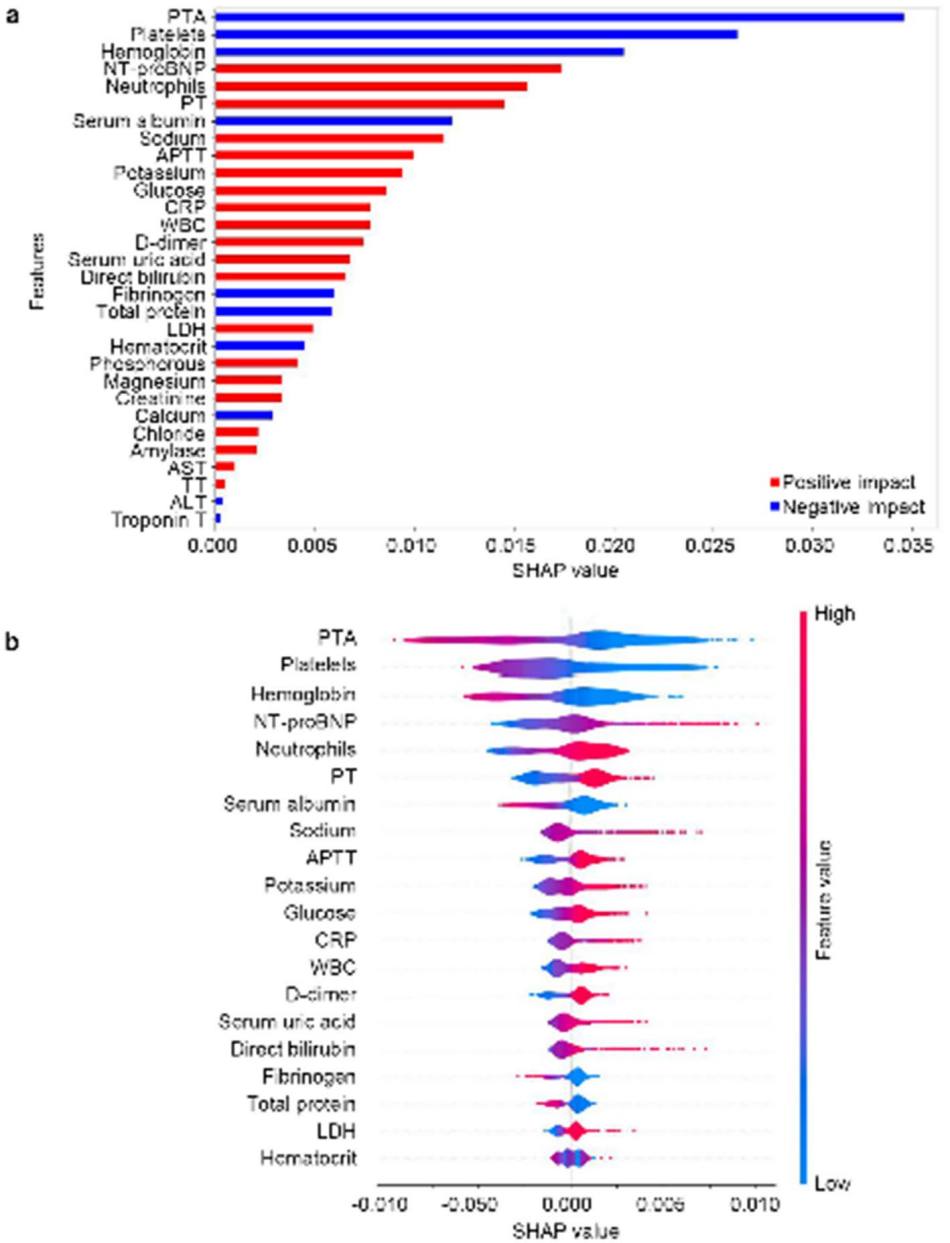
SHapley Additive exPlanations (SHAP) results. **a** SHAP feature importance; **b** SHAP summary plot of the top 20 variables. *PTA* prothrombin activity, *NT-proBNP* N-terminal pro-BNP, *PT* prothrombin time, *APTT* activated partial thromboplastin time, *CRP* C-reactive protein, *WBC* white blood cell, *LDH* lumbar disc herniation, *AST* aspartate aminotransferase, *TT* thrombin time, *ALT* alanine aminotransferase, *TnT* troponin T

As shown in Fig. 5a, the mean absolute SHapley Additive exPlanation (SHAP) value indicates individual feature importance in the ETC model, and the top ten variables were prothrombin activity (PTA), platelets (PLT), hemoglobin, N-terminal pro-BNP (NT-proBNP), neutrophils, prothrombin time (PT), serum albumin, sodium, activated partial thromboplastin time (APTT), and potassium. According to the summary plot (Fig. 5b), PTA, PLT, hemoglobin, and serum albumin were negatively correlated with IHCA occurrence. For example, a low PTA increases the importance of IHCA prediction, whereas a high PTA reduces the importance of IHCA prediction. In contrast, NT-proBNP, neutrophils, PT, sodium, APTT, and potassium were positively correlated with IHCA occurrence. The dependence plots of the SHAP value of top ten important variables are shown in Additional file 1: Fig. S1. The difference of abnormalities of top ten important variables between patients with and without IHCA is shown in Additional file 2: Table S2 [24–30]

## Discussion

In this study, we developed a machine learning-derived prediction model of IHCA in patients treated with rescue therapy. After fivefold cross-validation, the prediction model of IHCA based on the ETC algorithm showed the best performance among five algorithms used for model development. The SHAP interpreted the output of machine learning model and estimated the positive and negative contributions of each feature to the model prediction. The top ten important variables included PTA, PLT, hemoglobin, NT-proBNP, neutrophils, PT, serum albumin, sodium, APTT, and potassium, which are important predictors for IHCA in rescue-treated patients and provide valuable information for early intervention in rescue-treated patients to prevent IHCA.

ETC algorithm is an ensemble classifier that uses unpruned decision trees from the training datasets to construct an extremely randomized or extra tree classifier [31]. In this study, the ETC model showed excellent performance and better predictive power than the other four machine learning models. Notably, this model could predict the occurrence of IHCA in 24 h after testing laboratory parameters, and all variables were easy to obtain and under strict laboratory quality control. Therefore, this model is suitable for clinical practice in large tertiary hospitals.

Coagulopathy often occurs after resuscitation from cardiac arrest or during cardiac arrest [32]. Previous studies [33] have suggested that consumptive coagulopathy played a central role in the pathogenesis of cardiac arrest and the activation of the coagulation system was persistent during cardiac arrest. Among rescue-treated patients, sepsis-related coagulation dysfunction is one of the most common causes of death [34–36]. Because of the excessive production of plasminogen activator inhibitor-1, sepsis-related disseminated intravascular coagulation (DIC) causes excessive inhibition of fibrinolysis and may result in related prothrombotic effects, leading to reduced tissue perfusion, organ dysfunction, and poor outcomes [37, 38]. PT, APTT, and PLT are generally used to evaluate blood coagulation function, and have a high predictive value for DIC prediction [39]. Moreover, previous studies [40, 41] have indicated that PT prolongation and PLT decline are associated with increased mortality in patients with sepsis. Consistently, our study showed that coagulation markers are important predictors for IHCA. PT and APTT were positively correlated with the occurrence of IHCA, whereas PTA and PLT were negatively correlated with the occurrence of IHCA. 16.09% of patients with IHCA had abnormally prolonged PT by more than 3 s, and 42.20% of patients with IHCA had a PLT < 100 × 10^9^/L. However, clinicians always ignored coagulation disorders at an early stage, because these coagulation parameters were easily affected by multiple diseases and the fluctuation range is large [39, 42]. The findings of our study indicated that more attention should be paid to the progressive deterioration of coagulation parameters. It is necessary to monitor coagulation parameters in patients treated with rescue therapy and intervene coagulation disorders as early as possible.

As a commonly used infection index in clinics, neutrophils have been proven as biomarkers of sepsis [43, 44], which explains the finding that higher neutrophils was positively correlated with the occurrence of IHCA. Consistently, patients with cardiac arrest were frequently accompanied with infection, as shown in Additional file 1: Fig. S1. Albumin is the one of the most important components in human plasma, which can reflect the nutritional status and maintain osmotic pressure. Hofer et al. [45] found that the plasma albumin level in septic patients was significantly lower than that in non-septic patients. Our study found that serum albumin was negatively correlated with incident IHCA, which supports the effort of early management on nutritional status to prevent the occurrence of IHCA.

Previous studies [3] suggested that cardiovascular problems are the most common cause of cardiac arrest (50–60%). Higher brain natriuretic peptide is significantly associated with more severe cardiac injury and poorer prognosis. Pfister et al. [46]showed that NT‐proBNP was associated with both death and cardiovascular composite outcomes in a clinical population of patients with cardiovascular disease or chronic kidney disease. Similar to these findings, the SHAP results in our study showed that NT-proBNP is a strong predictor of IHCA and those with high NT-proBNP were more likely to suffer IHCA. In addition, 64.25% of patients with IHCA had an NT-proBNP level > 450 pg/mL, as shown in Additional file 2: Table S2.

Electrolyte disorder is one of the potential cause of cardiac arrest, among which potassium disorder is closely related to life-threatening arrhythmia [47]. Potassium is the main intracellular cation in the human body, which regulates the cardiac function, bones’ metabolism, and acid–base balance. Abnormality in potassium concentration can lead to serious complications. Patients with hyperkalemia are in extremely high risk of life-threatening cardiac arrest. Meanwhile, our study found that the level of sodium is positively correlated with the occurrence of IHCA in rescue-treated patients. A former study suggested that hypernatremia (> 145 mmol/L) can increase the risk of mortality [25]. Coppini et al. [48] found that the change in intracellular calcium homeostasis and the increase of late sodium current promoted arrhythmia. Additionally, Wu et al. [11] found that low level of hemoglobin is related to incident cardiac arrest. Taken together, these findings explain the importance of electrolyte and hemoglobin in the prediction of IHCA and suggest that the blood concentration of electrolyte and hemoglobin should be monitored frequently in rescue-treated patients to better prevent the occurrence of IHCA.

The major strength of our study is that we developed a reliable machine learning model to predict IHCA in rescue-treated patients solely using readily available laboratory parameters, which minimums the potential subjective bias that is common in self-reported data and medical texts, and enables our model to be more robust and applicable. This model promotes accurate prediction for IHCA in rescue-treated patients, which may further reduce the burden in frontline healthcare and improve the rescue success rate. However, several limitations should be considered. First, the major limitation of our study is that we only included patients from a single hospital, lacking external validation, and due to issues like missing data, we were unable to compare our results with the existing models. This may limit the generalizability of our model to other hospitals or regions. However, we only used objective laboratory test data in model development. The homogeneous nature of laboratory test data could help to reduce potential confounding due to region and health care disparities. Second, our study was based on a retrospective design, which may cause selection bias. Future prospective study and external validation are still warranted to further validate the model of our study.

## Conclusion

We developed an appreciable ETC model to predict IHCA in rescue-treated patients solely using routine laboratory parameters. The model showed that the major risk factors for IHCA in rescue-treated patients were PTA, PLT, hemoglobin, NT-proBNP, neutrophils, PT, serum albumin, sodium, APTT, and potassium. During the hospitalization of rescue-treated patients, physicians should attach great importance to frequently monitoring these parameters to prevent the occurrence of IHCA as possible.

## Methods

### Study population

This study was reviewed and approved by the ethics committee of the First Medical Center of Chinese PLA General Hospital (Ethics approval number: S2023-282-01). Rescue-treated patients were retrospectively identified from the hospital information system at the First Medical Center of the PLA General Hospital in Beijing, China, from January 2016 to December 2020. The inclusion criteria [11, 49, 50] were: (1) age ≥ 18 years and (2) length of hospital stay > 24 h; the exclusion criteria [11, 49, 50] were: (1) patients who had IHCA before rescue treatment, (2) patients who had history of out-of-hospital cardiac arrest (OHCA), (3) patients who had IHCA during surgery, and (4) patients with missing data on laboratory test.

### Prediction outcome

The primary outcome measure was the incidence of IHCA. This study defined IHCA as hospitalized patients whose pulse disappeared and required chest compression or defibrillation because of electrical defibrillation/cardioversion events. Only the first cardiac arrest that occurred in the hospital was analyzed [3, 49, 51].

### Candidate features

We abstracted data of the rescue-treated patients from the hospital information system. The following variables were collected: (1) demographic data: sex, age, body mass index (BMI), drinking, and smoking history; (2) basic diseases: hypertension and diabetes; (3) laboratory results: blood counts, biochemical markers, and coagulation markers, and C-reactive protein (CRP). For patients who experienced IHCA (positive samples), we collected laboratory data that were tested within the 24 h before incident IHCA. For patients who did not experienced IHCA (negative samples), we collected laboratory data that were tested within the 24 h after admission.

### Data processing

Candidate variables with missing values greater than 40% were excluded [52]. We calculated correlation coefficient between all variables and identified those pairs with high correlation coefficient (> 0.8). In the pair of variables with high correlation coefficient, the one with lower variable importance would be excluded. We discarded outliers of each variable, which were defined as values whose difference with mean was greater than threefold standard deviation. Missing values were handled using means substitution method [53, 54]. Finally, given that the negative samples are several times of positive samples in our study, we conducted undersampling using k-means clustering algorithm to balance the imbalanced data sets. The k-means algorithm recognizes each negative sample as an eigenvector and divides all negative samples into *n* eigenvector datasets with similar features, where *n* is the same with the number of positive samples. For each dataset, the k-means algorithm selects 1 eigenvector, which is the closest to the mean of the eigenvectors in the dataset, as a negative sample, and ultimately forms a dataset with *n* negative samples to create sample balance [55].

### Model development and validation

The dataset used for model development was randomly partitioned into two samples: 80% of the participants for model training and parameter learning and 20% of the participants for model performance evaluation and comparison. We used the ETC, logistic regression, random forest, SVM, and decision tree algorithms to develop a model for early prediction of IHCA. The performance metrics of the five models were evaluated by calculating the AUC of the receiver-operating characteristic curve, accuracy, specificity, sensitivity, and F1-score. Furthermore, we performed fivefold cross-validation to test the consistency and robustness of the model. To interpret the final predictive model, we used SHAP to explain the output of the model by evaluating the variable importance and the impact direction of variables [56]. Model development and validation were conducted using Python Version 3.8 (Python Software Foundation, Wilmington, DE, USA).

### Statistical analysis

Continuous variables with a normal distribution were presented as the mean ± standard deviation, and t tests were used for comparison between groups. Furthermore, non-normally distributed continuous variables were presented as median (interquartile range). Moreover, the Mann–Whitney *U* test was used for comparisons between groups. Categorical variables were presented as percentages (%), and the Chi-square test was used for comparisons between groups. To enhance the interpretability of our model, we provided the difference of abnormalities of parameters with high feature importance between patients with and without IHCA. All statistical analyses were conducted using SPSS statistical software (version 26.0; IBM Corp., Armonk, NY, USA). *P* value < 0.05 was considered statistically significant.

### Supplementary Information

**Additional file 1:** **Fig. S1.** SHapley Additive exPlanations (SHAP) dependence plot for the top 10 variables.** Fig. S2.** Admission diagnoses of In-hospital cardiac arrest.

**Additional file 2:** **Table S1.** Baseline characteristics of the undersampled dataset stratified by incident IHCA. **Table S2.** The difference of abnormalities of top 10 important parameters between patients with and without IHCA.

## Data Availability

The data sets are available from the corresponding author.

## Abbreviations

IHCA: In-hospital cardiac arrest
CA: Cardiac arrest
OHCA: Out-of-hospital cardiac arrest
BMI: Body mass index
CRP: C-reactive protein
SVM: Support vector machine
ETC: Extra trees classifier
AUC: Area under the receiver operator curve
SHAP: SHapley Additive Explanations
PTA: Prothrombin activity
PLT: Platelet
NT-proBNP: N-terminal pro-BNP
PT: Prothrombin time
APTT: Activated partial thromboplastin time
DIC: Disseminated intravascular coagulation
