# Supplementary material for: Development of early prediction model of in-hospital cardiac arrest based on laboratory parameters
Source: Biomed Eng Online. 2023 Dec 6;22:116. doi: 10.1186/s12938-023-01178-9 (PMC10698946; doi:10.1186/s12938-023-01178-9)
Supplement: Supplementary file 1 — Additional file 1: Fig. S1. SHapley Additive exPlanations (SHAP) dependence plot for the top 10 variables. Fig. S2. Admission diagnoses of In-hospital cardiac arrest. [file 12938_2023_1178_MOESM1_ESM.docx]

**Additional Information**


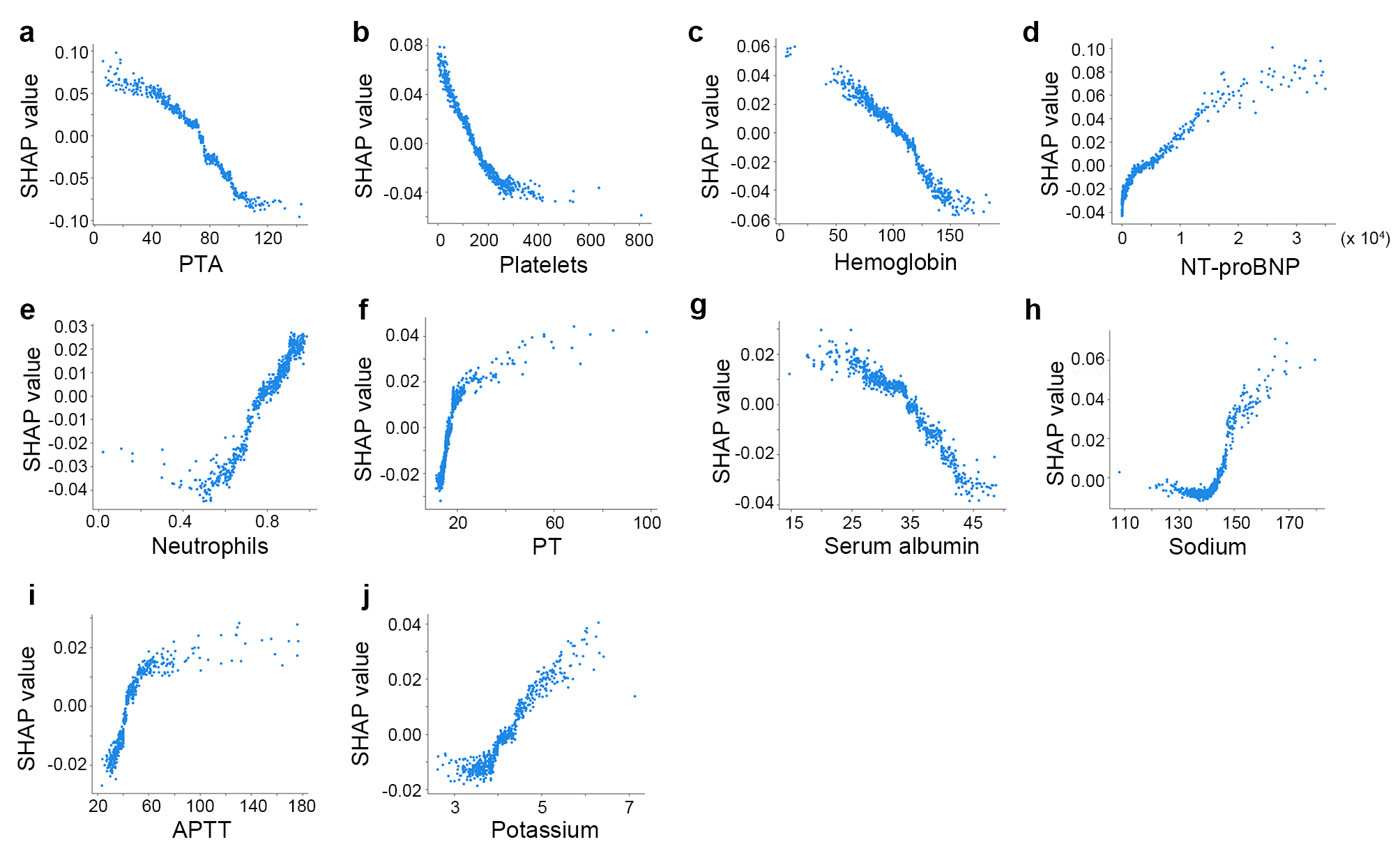


**Fig. S1** SHapley Additive exPlanations (SHAP) dependence plot for the top 10 variables. a. The level of PTA is negatively correlated with the occurrence of cardiac arrest in critically ill patients. b. The level of PLT is negatively correlated with the occurrence of cardiac arrest in critically ill patients. c. The level of hemoglobin is negatively correlated with the occurrence of cardiac arrest in critically ill patients. d. The level of NT-proBNP is positively correlated with the occurrence of cardiac arrest in critically ill patients. e. The level of neutrophils is positively correlated with the occurrence of cardiac arrest in critically ill patients. f. The level of PT is positively correlated with the occurrence of cardiac arrest in critically ill patients. j.The level of serum albumin is negatively correlated with the occurrence of cardiac arrest in critically ill patients. h. The level of sodium is positively correlated with the occurrence of cardiac arrest in critically ill patients. i. The level of APTT is positively correlated with the occurrence of cardiac arrest in critically ill patients. j. The level of potassium is positively correlated with the occurrence of cardiac arrest in critically ill patients.

**Abbreviations:** PTA, prothrombin activity; NT-proBNP, N-terminal pro-BNP; PT, prothrombin time; APTT, activated partial thromboplastin time.

**
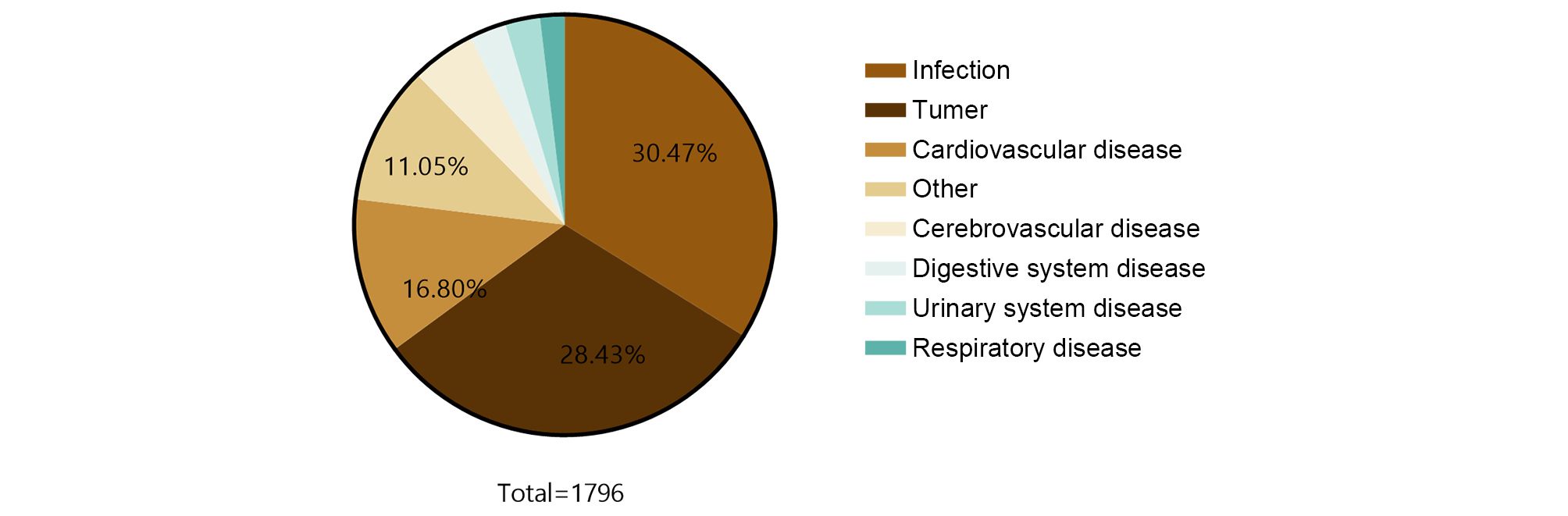
**

**Fig. S2** Admission diagnoses of In-hospital cardiac arrest.
