# Supplementary material for: Development of early prediction model of in-hospital cardiac arrest based on laboratory parameters
Source: Biomed Eng Online. 2023 Dec 6;22:116. doi: 10.1186/s12938-023-01178-9 (PMC10698946; doi:10.1186/s12938-023-01178-9)
Supplement: Supplementary file 2 — Additional file 2: Table S1. Baseline characteristics of the undersampled dataset stratified by incident IHCA. Table S2. The difference of abnormalities of top 10 important parameters between patients with and without IHCA. [file 12938_2023_1178_MOESM2_ESM.docx]

**Additional Information**

**Table S1**: **Baseline characteristics of the undersampled dataset stratified by incident IHCA**

| **Characteristic Mean (SD) or N (%)** | **Non-IHCA (n=1796)** | **IHCA(n=1796)** | **P-value** |
| --- | --- | --- | --- |
| Age, years | 63 (50-73.25) | 80 (65-87.25) | <0.001 |
| Male | 1131 (62.97) | 1162 (64.70) | 0.282 |
| BMI, kg/m2 | 24 (21-27) | 22 (19-25) | <0.001 |
| Smoking | 527 (29.34) | 416 (23.16) | <0.001 |
| Drinking | 503 (28.01) | 342 (19.04) | <0.001 |
| **Complications** |  |  | <0.001 |
| Hypertension | 729 (40.59) | 872 (48.55) | <0.001 |
| Diabetes | 379 (21.10) | 464 (25.84) | 0.001 |
| **Laboratory results** |  |  | <0.001 |
| D-Dimer, ug/ml | 1.31 (0.46.2.94) | 3.51 (2.03-7.11) | <0.001 |
| Sodium, mmol/L | 140.3 (137.6-142.6) | 142.2 (136.8-148.8) | <0.001 |
| White blood cell count, 10^9^/L | 7.32 (5.43-10.35) | 11.9 (7.34-17.71) | <0.001 |
| Direct bilirubin, umol/L | 3.9 (2.5-6.88) | 10.8 (5-34.4) | <0.001 |
| PT, s | 14 (13.2-15.3) | 19.1 (16.1-24.4) | <0.001 |
| PTA, % | 86 (73-97) | 50 (35-67) | <0.001 |
| TT, s | 15.9 (15-16.8) | 17.1 (15.5-20.55) | <0.001 |
| Phosphorous, mmol/L | 1.07 (0.88-1.25) | 1.19 (0.83.1.72) | <0.001 |
| Creatinine, umol/L | 76 (60.9.97.4) | 132.2 (75.7-230.3) | <0.001 |
| Lumbar disc herniation, U/L | 190.45 (153.5-295.15) | 391.35 (250.88-741.48) | <0.001 |
| Alanine aminotransferase, U/L | 17.6 (11.3-33.5) | 20.65 (9.3-56.98) | <0.001 |
| Potassium, mmol/L | 3.92 (3.64-4.23) | 4.28 (3.81-4.85) | <0.001 |
| NT-proBNP, pg/ml | 474.8 (125.22-1808.25) | 5006 (1462.75-12468.25) | <0.001 |
| Magnesium, mmol/L | 0.85 (0.79.0.92) | 0.88 (0.75-1) | <0.001 |
| Glucose, mmol/L | 6.12 (4.96-8.36) | 8.56 (6.47-12.07) | <0.001 |
| Platelets, 10^9^/L | 197 (151-253) | 92 (40-167) | <0.001 |
| Amylase, U/L | 53.6 (38.2-79.4) | 71.2 (38.98-136.5) | <0.001 |
| Hemoglobin, g/L | 120 (98.136) | 87 (73-104) | <0.001 |
| Calcium, mmol/L | 2.17 (2.03-2.27) | 8.56 (6.47-12.07) | <0.001 |
| APTT, s | 37.8 (34.2-42.5) | 49.4 (42-62.08) | <0.001 |
| Hematocrit, L/L | 0.35 (0.29-0.4) | 0.26 (0.22-0.32) | <0.001 |
| Fibrinogen, g/L | 3.56 (2.83-4.58) | 2.92 (1.9-4.18) | <0.001 |
| Neutrophils | 0.73 (0.63.0.86) | 0.88 (0.8-0.93) | <0.001 |
| Total protein, g/L | 64.2 (57.9-69.45) | 57.1 (51.1.63.3) | <0.001 |
| Troponin T, ng/ml) | 0.02 (0.01-0.12） | 0.11 (0.05-0.28) | 0.119 |
| Serum uric acid, umol/L | 302.1 (229-05-391.45) | 387.9 (249.55-562.35) | <0.001 |
| Chloride, mmol/L | 103.2 (100.3-106) | 102.4 (97-108.7) | 0.050 |
| Serum albumin, g/L | 36.9 (32.2-40.5) | 31 (27.4-34.7） | <0.001 |
| Aspartate aminotransferase, U/L | 20.1 (14.5-40.25) | 42.95 (21-125.45) | <0.001 |
| CRP, mg/dl | 1.3 (0.2-5.84) | 6.77 (3.03-12.13) | <0.001 |

**Abbreviations:** BMI, Body Mass Index; PT, prothrombin time; PTA, prothrombin activity; TT, thrombin time; NT-proBNP, N-terminal pro-BNP; APTT, activated partial thromboplastin time; CRP, C-reactive protein.

**Table S2**: **The difference of abnormalities of top 10 important parameters between patients with and without IHCA**

| **Features** | **Range** | **Non-IHCA**  **(n=1796)** | **IHCA**  **(n=1796)** | **P-value** |
| --- | --- | --- | --- | --- |
| **PTA** | <40% | 34(1.89%) | 341(18.99%) | <0.001 |
| **platelets** | ＜100× 10^9^/L | 142(7.91%) | 758(42.20%) | <0.001 |
| **Hemoglobin** | Female, <120g/L | 381(21.21%) | 496(27.62%) | <0.001 |
|  | Male, <130g/L | 427(23.78%) | 928(51.67%) | <0.001 |
| **NT-proBNP** | ＞450pg/mL | 567(31.57%) | 1154(64.25%) | <0.001 |
|  | ＞125pg/mL | 841(46.83%) | 1230(68.49%) | <0.001 |
| **Neutrophils** | >0.7 | 1346(74.94%) | 933(51.95%) | <0.001 |
| **PT** | prolong≥3s | 26(1.45%) | 289(16.09%) | <0.001 |
| **Serum albumin** | <30g/L | 263(14.64%) | 622(34.63%) | <0.001 |
| **Sodium** | >145mmol/L | 140(7.80%) | 618(34.41%) | <0.001 |
| **APTT** | prolong≥10s | 372(20.71%) | 769(42.82%) | <0.001 |
| **Potassium** | >5.5mmol/L | 6(0.33%) | 101(5.62%) | <0.001 |

**Abbreviations:** PTA, prothrombin activity; NT-proBNP, N-terminal pro-BNP; PT , prothrombin time; APTT, activated partial thromboplastin time.
